# A Survey of Didemnin Depsipeptide Production in *Tistrella*

**DOI:** 10.3390/md21020056

**Published:** 2023-01-17

**Authors:** Robert J. Stankey, Don Johnson, Brendan M. Duggan, David A. Mead, James J. La Clair

**Affiliations:** 1 Terra Bioworks Inc., Middleton, WI 53562, USA; 2 Skaggs School of Pharmacy and Pharmaceutical Sciences, University of California, 9500 Gilman Drive, San Diego, CA 92093-0657, USA; 3 Department of Chemistry and Biochemistry, University of California, San Diego, CA 92093-0358, USA; 4 Xenobe Research Institute, P.O. Box 3052, San Diego, CA 92163-1052, USA

**Keywords:** didemnin, depsipeptide, non–ribosomal peptide, biosynthesis, metabolomics

## Abstract

As one of the first families of marine natural products to undergo clinical trials, the didemnin depsipeptides have played a significant role in inspiring the discovery of marine drugs. Originally developed as anticancer therapeutics, the recent re-evaluation of these compounds including synthetically derived dehydrodidemnin B or Aplidine, has led to their advancement towards antiviral applications. While conventionally associated with production in colonial tunicates of the family *Didemnidae*, recent studies have identified their biosynthetic gene clusters from the marine–derived bacteria *Tistrella mobilis*. While these studies confirm the production of didemnin X/Y, the low titer and general lack of understanding of their biosynthesis in *Tistrella* currently prevents the development of effective microbial or synthetic biological approaches for their production. To this end, we conducted a survey of known species of *Tistrella* and report on their ability to produce the didemnin depsipeptides. These data were used to develop conditions to produce didemnin B at titers over 15 mg/L.

## 1. Introduction

**Figure 1:**
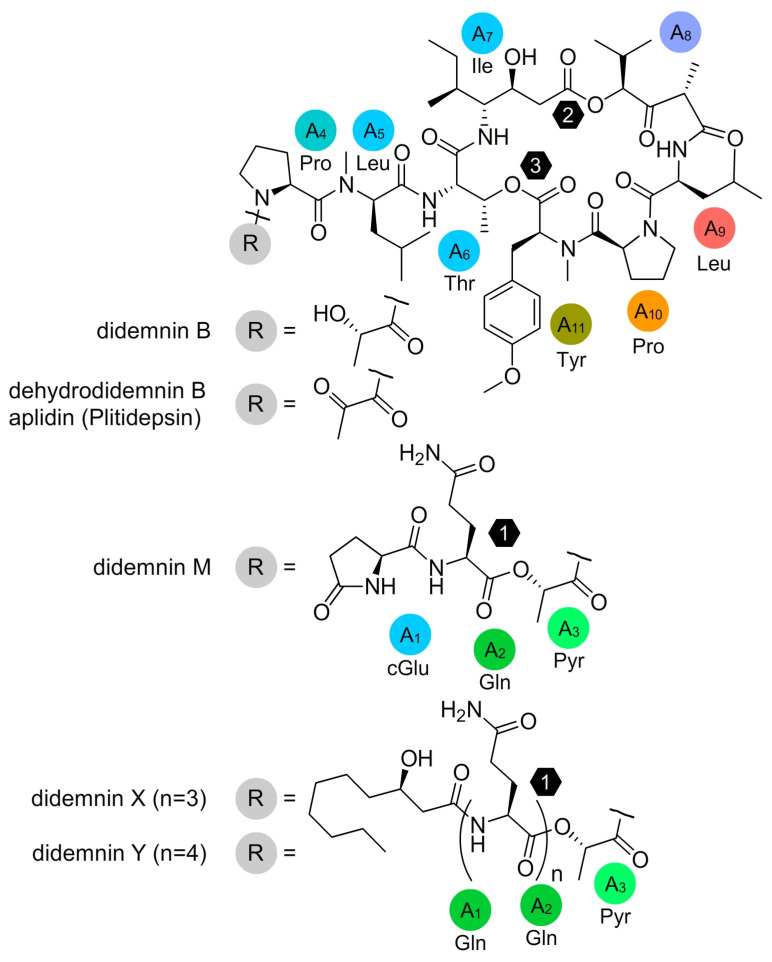
Structures of select didemnin depsipeptides including aplidine and didemnin B, M, X and Y. Structures are provided with their biosynthetic adenylation (A) domain and amino acid selectivity [17]. The numbers within the hexagons indicate the positions of each ester linkage.

First reported in 1981 [1], the didemnin depsipeptides, including the tamandarins [2], represent a unique family of non-ribosomal-produced peptides comprising two or three ester-linked peptides and/or lipids (Figure 1). Among this family, didemnin B and M have demonstrated potent in vitro cytotoxicity [3,4] and in vivo antitumor activity [5] resulting in its translation into clinical trials [6,7,8] against adenocarcinoma of the kidney, advanced epithelial ovarian cancer, and metastatic breast cancer. While initially unexplored, early screening efforts also identified potent antiviral activity against DNA/RNA viruses [9]. Recently, dehydrodidemnin B (Aplidine, Plitidepsin) [7] was shown to have potent activity against the severe acute respiratory syndrome corona-virus 2 (SARS-CoV-2) in vitro, in animal models [10,11,12] and in humans [13].

In 2011, a group at the Yakult Central Institute reported the identification of didemnin B from a marine-derived *Tistrella mobilis* strain [14]. The genus *Tistrella* was first identified from waste water in Thailand and reported by Shi et al. in 2002 [15], with a second species, *T. bauzanensis*, identified in 2011 by Zhang et al. from soil in a heavy-metal-contaminated site in Italy [16]. Thus, until the report by Tsukimoto et al. [14], the only record of this genus came from terrestrial sources, albeit aqueous. A year later, a group from Hong Kong working closely with a team at Scripps Institution of Oceanography and University of California, San Diego, published the complete biosynthetic pathway to didemnin X/Y from a free-living *Tistrella mobilis* strain isolated from the Red Sea [17]. In addition to this pathway, they also used MALDI-imaging to demonstrate in real-time the production of didemnin X/Y as the microbe was growing. Thus, there was absolutely no question that this free-living microbe produced the didemnin depsipeptides.

While this compound class has been known to possess potent antiviral activity, only its anti-leukemic activity has been explored clinically until recently [6,7,8,10]. Unfortunately, limited availability of these natural products (currently obtained by isolation from marine tunicates or through total synthesis) has hindered their study and clinical exploration. Recent studies suggest that microbial production of the didemnins from two cultures of their native *Tistrella* sp. producers yields predominantly uncoupled partial products with low levels (<1 mg/L) of the desired didemnins [14,17]. This evidence not only suggests that microbial culturing presents even further challenges, but also indicates that without the development of heterologous production systems these natural products and their derivatives will remain cost-ineffective for the treatment of viral infections such as SARS-CoV-2 [10,11,12,13].

Although the discovery of a microbial origin for the didemnin depsipeptides suggests a viable means of natural or synthetic biological production, ameliorating the lack of understanding of the biosynthetic potential of *Tistrella* is critical to its ultimate success. Since the first description in 2002 [15], only a handful of studies have explored the biosynthetic potential of *Tistrella*; these include recent efforts to evaluate the biosynthetic gene clusters (BGCs) associated with 3–thia–α–amino acid [18], thalassospiramide [19,20], and the didemnin depsipeptides [17]. Here, we apply an algorithm-based approach to evaluate the metabolomic profile of cultures from three strains of *Tistrella*.

## 2. Results

### 2.1. Establishing Viable Culture Conditions

*Tistrella* are alphaproteobacteria belonging to the Rhodospirillaceae family [15]. Three strains of *Tistrella* were examined in this study: *Tistrella mobilis* JCM21370 [15], *Tistrella bauzanensis* BZ78 (DSM22817) [16], and *Tistrella mobilis* YIT12409 [14]. We began by identifying media and conditions to culture them. We found that *T. mobilis* JCM21370, *T. mobilis* YIT12409, and *T. bauzanensis* DSM22817 all grew readily on 74NA Petri dishes at 30 °C for the *mobilis* strains and 25 °C for the *bauzanensis* strain. In order to test antibiotic resistance in *T. mobilis* JCM21370 and *T. mobilis* YIT12409, we picked single colonies to 14 mL round-bottom polystyrene tubes containing 3 mL 74NB. After 3 days at 30 °C with 200 RPM shaking, 1 µL was transferred to culture tubes containing fresh 3 mL 74NB and each of the following antibiotics (Table 1): apramycin (50 µg/mL), carbenicillin (50 µg/mL), chloramphenicol (25 µg/mL), colistin (5 µg/mL), kanamycin (50 µg/mL), hygromycin (100 µg/mL), and nalidixic acid (30 µg/mL). After 3 days at 30 °C with 200 RPM shaking, we compared the growth relative to cultures without any added antibiotic. *T. mobilis* JCM21370 was resistant to carbenicillin and colistin with no apparent growth defect, while some resistance to nalidixic acid was observed with very weak growth. *T. mobilis* YIT12409 was only resistant to carbenicillin.

### 2.2. Comparative Analyses for the Production of Didemnin Depsipeptides

Strains were cultured uniformly for didemnin expression to produce the samples given in Table 2 in the following way: From a frozen (−80 °C) glycerol stock of each strain, we streaked to 74NA Petri dishes and incubated at either 25 °C (*T. bauzanensis*) or 30 °C (*T. mobilis*) until well-formed colonies appeared. For 100 mL- to 200 mL-scale cultures, a single colony was picked to 250 mL glass conical flasks containing 100 mL of media and incubated with 200 RPM shaking according to the conditions given in Table 2. For 1 L to 5 L cultures (used to test scaled up isolation), a single colony was picked to 100 mL of 74NB, incubated with 200 RPM shaking at either 20 °C for 5 days (*T. bauzanensis*) or 30 °C for 3 days (*T. mobilis*). Then, 1 mL was transferred to 2 L glass conical flasks containing 1 L of media and incubated with 200 RPM shaking according to the conditions given in Table 2. Cultures developed from this study were extracted and evaluated using NMR (Section 2.3) and atomic sort methods (Section 2.3).

### 2.3. NMR Data Collection

Aliquots (5–10% of total sample) from each crude extract (see Materials and Methods for preparation in Section 2.2) were dried by N_2_ flow and dissolved in 200 µL of CD_3_OD. A 50 µL aliquot of this sample was added to a 1.7 mm NMR SampleJet (Bruker) NMR tube. NMR data were acquired with a Bruker Avance III 600 MHz spectrometer equipped with a 1.7 mm cryoprobe. Chemical shifts were referenced using the corresponding solvent signals (δ_H_ 3.31 and δ_C_ 49.0 for CD_3_OD). The NMR spectra were processed using MestReNova 12.0.3 (Mestrelab Research) or TopSpin 3.6 (Bruker Biospin) software.

### 2.4. Atomic Sort Metabolomics Analysis

Comparison of a database of ^1^H–^13^C HSQC-peak lists against a peak list of each *Tistrella* extract allowed for identification of the components of each culture. The peaks of each compound in the database were matched to the closest peak in each culture spectrum using the Atomic Sort algorithm [21]. This algorithm calculates the Euclidean distance from a query peak to the closest peak in a reference set. The ^1^H and ^13^C dimensions are normalized by dividing by the range of chemical shifts for each dimension in the reference set, thus expressing the distance between two peaks as a fraction of the total chemical shift space. Calculating Atomic Sort distances (*d_AS_*) between query and reference peak lists, then taking the median $\tilde{d_{AS}}$ gives a quantitative measure of how well the spectra match. Repeating this for thousands of compounds, then sorting on $\tilde{d_{AS}}$ enables automated and objective compound identification. Matches to a subset of a reference compound’s peaks indicates the presence of a compound similar, but not identical, to one in the reference set.

**Figure 2:**
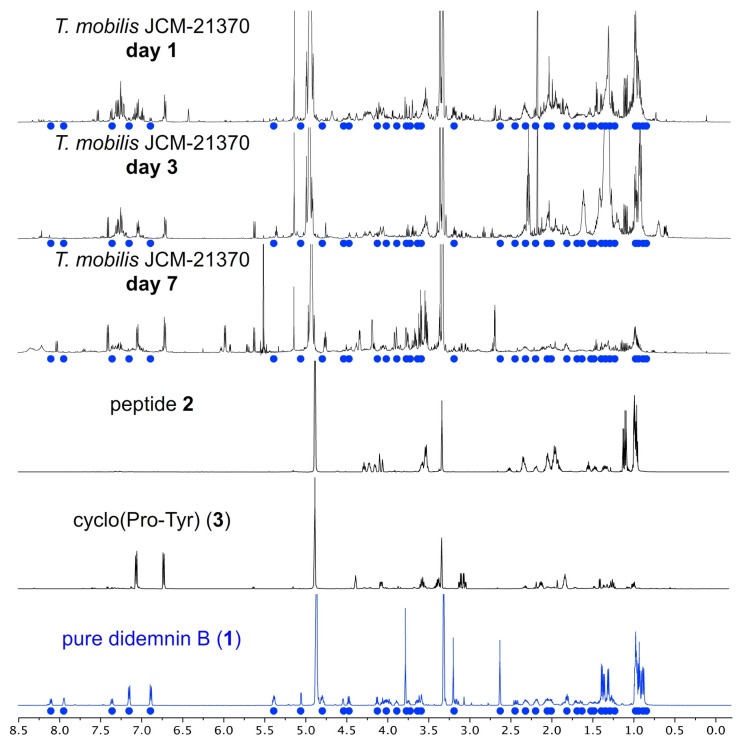
^1^H NMR spectra from a time course study on the growth of *T. mobilis* JCM21370. Didemnin B (**1**) could be detected in these cultures, as illustrated by comparing the NMR spectra of the extracts (black) to that of pure **1** (blue). Didemnin B (**1**) was isolated in a pure state after fractionation and chromatographic purification. Peaks from **1** (blue dots) are shown on each extract. NMR spectra of the **2** and cyclo(Pro–Tyr) (**3**) also isolated from *T. mobilis* JCM21370 are provided. Data from additional extracts (K-R, Table 2), including *T. mobilis* YIT12409, are provided within the Supplementary Information.

**Figure 3:**
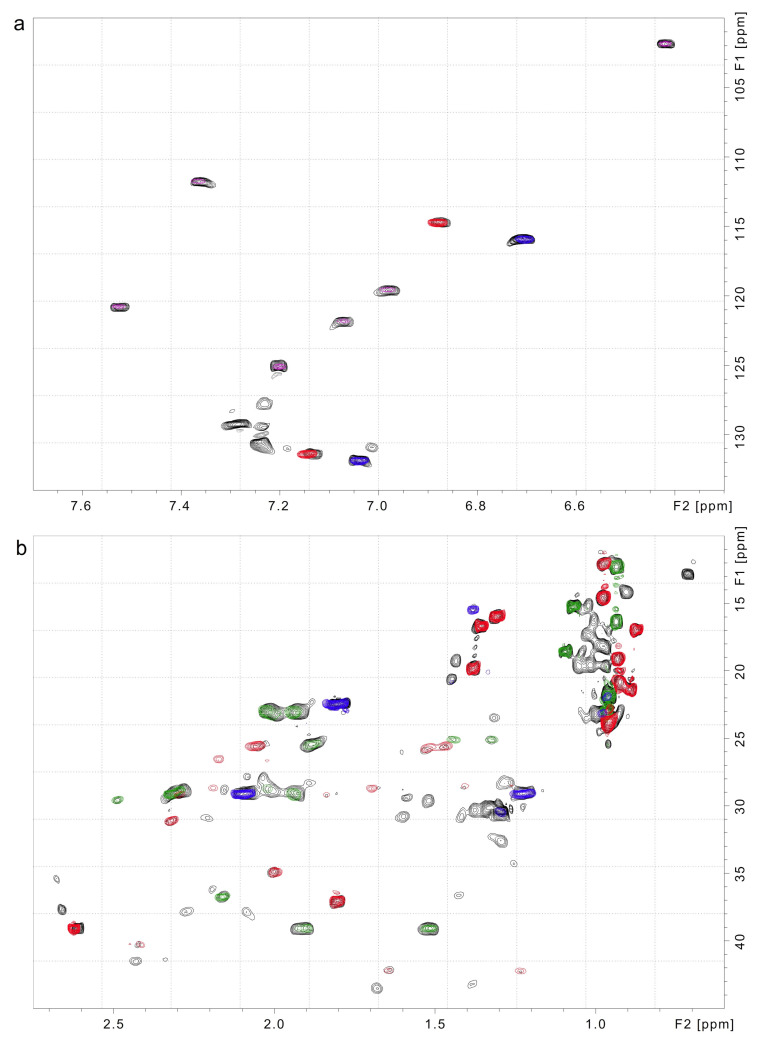
Atomic Sort identification of didemnin B (**1**) in the *T. mobilis* JCM21370. Expansions of (**a**) the aromatic and (**b**) the methyl regions of the ^1^H–^13^C HSQC spectrum of the *T. mobilis* JCM21370 extract H (black) overlaid with spectra of didemnin B (**1**, red), cyclo(Pro–Tyr) (**3**, blue), Leu–Pro–Ile–Pro–Ile (**2**, green), and indole (magenta). All peaks of these metabolites are observed in the *T. mobilis* JCM21370 extract spectrum, along with additional weaker peaks from metabolites that were not identified. Extract H (Table 2) was used for this study.

Didemnin B (**1**) was detected in cultures of both *T. mobilis* strains (Figure 2 and Figure 3, Table 3 and Table 4, and additional spectral data within the Supplementary Information). The *T. bauzanensis* DSM2281 strain did not produce detectable amounts of the depsipeptide. All three strains produced indole and short peptides, including cyclo(Pro–Tyr) (**3**, Figure 2), LPIPI (Leu–Pro–Ile–Pro–Ile, **2**), and a peptide that was not conclusively identified but is closely related to cyclo(Ile-Pro-Leu-Pro). Additionally, the solvent used for the NMR analysis (CD_3_OD) and n–hexane, or a long alkyl chain compound—a common impurity—was detected. Table 3 shows the $\tilde{d_{AS}}$ and the fraction of matched peaks for the identified metabolites. A smaller $\tilde{d_{AS}}$ indicates a better match and more confidence that the metabolite is present. Values of less than 0.1 indicate the presence of the compound, values of 0.1 to 0.2 suggest a related compound, and higher values are non-specific matches. Of the 200–250 peaks in each extract’s ^1^H–^13^C HSQC spectrum, ~50% could be assigned (Figure 3). Full Atomic Sort analyses of *T. mobilis* JCM21370, *T. mobilis* YIT12409 and *T. bauzanensis* DSM22817 are provided in the Supplementary Information.

The amount of each metabolite produced was quantified using integrals from the 1D ^1^H spectra and the residual CD_3_OD methyl resonance as an internal standard. The ^1^H–^13^C HSQC spectra were used to identify peaks for each metabolite with minimal overlap in the ^1^H spectra. Table 4 lists concentrations and masses of the metabolites identified in the extracts.

### 2.5. Didemnin BGC Architecture Analysis

**Figure 4:**
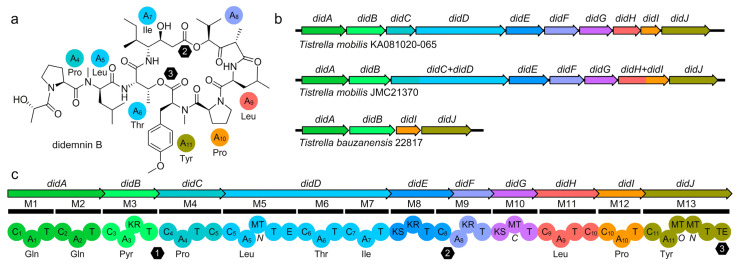
Didemnin biosynthetic gene clusters (BGCs). (**a**) Structure of didemnin B (**1**) with the A domain amino acid loading (colored spheres) and positions of thioester coupling (hexagons). (**b**) Didemnin BGCs from three different strains of *Tistrella*. The BGCs identified in each strain contain unique combinations of didA–didJ with domain module contraction in *T. mobilis* JCM21370 when compared to that observed in *T. mobilis* KA081020–065 BGC [17]. The non-producing BGC in *T. bauzanensis* was truncated including only the first and last two genes. (**c**) Didemnin BGC identified from *T. mobilis* KA081020–065 with domain (spheres) and module (bar) architecture shown. One of the two TE domains was observed outside of the BGC (Orf4, not shown). The numbers within the hexagons indicate the positions of each ester linkage.

Whole-genome sequence analysis was used to elucidate the presence and architecture of the putative didemnin BGCs in each of the four *T. mobilis* strains. The pathways from *T. mobilis* JCM21370 and *T. mobilis* YIT12409 contained didA–didJ (Figure 4) as elucidated in *T. mobilis* KA081020–065 [17]. Not surprisingly, the non-didemnin-producing *T. bauzanensis* DSM22817 strain was missing didC–didH and therefore lacked the machinery to install all but the Pro–Tyr motif, which could be attributed to the production of cyclo(Pro–Tyr) (**3**). While intact, profound differences were observed between each pathway where the BGC in *T. mobilis* JCM21370 contained didC–didD and didH–didI as a fusion within a single gene, as compared to *T. mobilis* KA081020–065. While an active didemnin producer, the GC-rich BGC from *T. mobilis* YIT12409 is being resequenced to confirm the organization of this GC rich cluster.

## 3. Discussion

In a detailed metabolomic analysis of extracts, we observed the production of didemnin B (**1**) by both *T. mobilis* strains, but not *T. bauzanensis* (Xu [17] observed didemnin B from *T. bauzanensis* TIO7329). NMR estimations indicate that these cultures contained ~30 mg/L of **1**. In practice, we were able to isolate 15.9 mg of pure didemnin B (**1**) (see NMR spectral data set in the Supplementary Information), providing an isolated yield of 7.9 mg/L. In prior studies [14,17], the low yield of didemnins was complicated by the fact that the crude extracts contained chemical shifts comparable to that of didemnin B, with the exception of the two ester protons between 4.9 and 5.5 ppm. In our studies, we only observed didemnin B (**1**), and the formation of other didemnin analogues such as didemnin X/Y was not detected. This simplifies compound purification and allows access to a single didemnin depsipeptide, **1**. Such observations further support the theory [17] that additional esterification in didemnin X/Y is transitory, likely as didemnin B is catabolized (or hydrolyzed) upon extended culturing.

This production was then screened across the two *T. mobilis* strains JCM21370 and YIT12409 using three different media. As shown in Table 5, **1** was produced by both strains in all three media evaluated. The highest titers were obtained from cultures of *T. mobilis* JCM21370 in 74NB media (K and M, Table 5), with titers predicted as high as 111.7 mg/L (M, Table 5). While not described in depth, the key to providing reproducible yields of **1** arose from careful regulation of the temperature and shaking, as well as conducting the growth in a relatively short period (3 days).

Meticulous isolation efforts enabled the other primary materials within extracts of culture H to be identified (Table 2). We were able to isolate sufficiently pure samples to identify two of the compounds as cyclo(Pro–Tyr) (**3**) and cyclo(Pro–Phe) (**4**) as validated by HRMS–ESI–MS for **3** with *m/z* calcd. for C_14_H_17_N_2_O_3_ [M+H]^+^ of 261.1234 and 261.1237 found and **4** with HRMS–ESI–MS with *m/z* calcd. for C_14_H_17_N_2_O_2_ [M+H]^+^ of 245.1285 and 244.1287 found. We also isolated samples of **2** (^1^H NMR spectrum in Figure 2), which was tentatively assigned as Leu-Pro-Ile-Pro-Ile. Fragments of didemnin B (**1**) were not detected, and the isoleucine containing peptides were distinguished from isostatine.

## 4. Materials and Methods

### 4.1. Strains

*Tistrella mobilis* JCM21370 was acquired from the Japan Collection of Microorganisms, RIKEN BioResource Center. *Tistrella mobilis* YIT12409 was received as a gift from Moriya Tsukimoto (Yakult Central Institute). *Tistrella bauzanensis* DSM22817 was acquired from the DSMZ—German Collection of Microorganisms and Cell Cultures.

### 4.2. Culture Conditions

*T. mobilis* JCM21370 and *T. mobilis* YIT12409 were streaked to 74 Nutrient Agar (74NA; peptone 5 g/L, beef extract 3 g/L, agar 15 g/L) and incubated for 2–3 days at 30 °C until colonies appeared. For liquid cultures, a single colony was picked to 74 Nutrient Broth (74NB) and incubated for 3 days at 30 °C with 200 RPM shaking. *T. bauzanensis* DSM22817 was streaked to 74NA and incubated for 3–5 days at 25 °C. For liquid cultures, a single colony was picked to 74NB and incubated for 5 days at 20 °C with 200 RPM shaking. Additional samples were obtained by culturing the strains in the following broths: Tryptic Soy Broth (TSB, Research Products International), GYP (glucose 10 g/L, yeast extract 4 g/L, peptone 2 g/L, Instant Ocean 17 g/L), and Seawater-Based Medium [14] (SBM; galactose 1 g/L, gelatin peptone 1 g/L, glycerol 1 g/L, yeast extract 1.5 g/L, peptone 5 g/L, Instant Ocean 30 g/L).

### 4.3. Extraction

Cultures were frozen and thawed prior to extraction. A common method was used for all extractions. As an example, frozen culture broth from *T. mobilis* JCM21370 (1 L) grown in 74NB for 3 days at 37 °C with shaking at 200 RPM was warmed to 23 °C, saturated with NaCl and added to a 2 L Squibb separatory funnel (2 L). The mixture was extracted with EtOAc (3 × 1 L), washed with brine (500 mL), and dried on Na_2_SO_4_. The solvent was removed by rotary evaporation using a round bottom flask (5 L). The contents of the flask were transferred to a 20 mL scintillation vial (VWR) by sequential washing of the flask with 3:1 EtOAc/MeOH and the solvent removed by rotary evaporation. Samples were stored at −20 °C. An aliquot (1–3%, weighed after transfer for each sample) was removed from each sample, dried in a ½ dram V1235CTFE TFE-lined vial (Glass Vials Inc., Hanover, MD, USA) and dried using N_2_ flow for NMR analyses as described in Section 4.4.

### 4.4. NMR Analyses

Aliquots from each extract in Section 4.3 dried by N_2_ flow were dissolved in 200 µL of CD_3_OD, and 50 µL of this sample was added to a 1.7 mm NMR SampleJet (Bruker) NMR tube. NMR data were acquired with a Bruker Avance III 600 MHz spectrometer equipped with a 1.7 mm cryoprobe. HSQC spectra were acquired using the Bruker pulse sequence “hsqcedetgpsisp2.3” modified to include a DIPSI-2 spinlock in the relaxation delay for the ASAP protocol [22]. Chemical shifts were referenced using the corresponding solvent signals (δ_H_ 3.31 and δ_C_ 49.0 for CD_3_OD). The NMR spectra were processed using Mnova 12.0.3 (Mestrelab Research) or TopSpin 3.6 (Bruker Biospin).

### 4.5. Purification of Didemnin B

A two-step procedure was used to purify didemnin B (**1**) from extract H (Table 2), which included fractionation followed by subsequent purification. The entire crude extract (524.2 mg) was dissolved in 5 mL of 9:1 CH_2_Cl_2_/MeOH and applied to a 20 cm × 2.5 cm flash column charged with SiliaFlash Irregular Silica Gel P60—40–63 µm mesh, grade 60 (SiliaCycle)—loaded and washed with hexanes. Fractions were collected by the sequential passage of 500 mL of hexanes (fraction A), 2:1 hexanes/EtOAc (fraction B), 1:1 hexanes/EtOAc (fraction C), EtOAc (fraction D), 4:1 EtOAc/MeOH (fraction E), 2:1 EtOAc/MeOH (fraction F), 1:1 EtOAc/MeOH (fraction G), and MeOH (fraction H). A 10 mL aliquot was taken from each fraction, dried with N_2_ flow and subjected to NMR analysis. Fraction D (42.5 mg) contained **1**. All fractions were saved by solvent removal using rotary evaporation followed by transfer to a 20 mL vial (Qorpak) by sequential washing of the flask with 3:1 EtOAc/MeOH and drying the solvent using rotary evaporation. Fraction D was then subjected to a second purification using high quality SiliaFlash 25 µm spherical Silica Gel with a gradient of hexanes to 1:9 hexanes/acetone providing 22.8 mg of **1** (~85% purity), which upon repetition (third purification) provided 15.9 mg of **1** (purity ≥ 98%).

### 4.6. Genome Sequencing

Single colonies of *T. mobilis* JCM21370 were picked and cultured in 100 mL 74NB for 3 days at 30 °C with 200 RPM shaking. The cultures were centrifuged at 5000× *g* for 15 min. The supernatants were discarded and the pellets frozen at –80 °C. The pellets were shipped with dry ice to the Microbial Genome Sequencing Center (Pittsburgh, PA, USA) for gDNA extraction and sequencing. The gDNA samples were sequenced with a combination of Illumina and Oxford Nanopore methods. Quality control and adapter trimming were performed with bcl2fastq [23] and porechop [24] for Illumina and ONT sequencing, respectively. Hybrid assembly with Illumina and ONT reads was performed with Unicycler [25]. The genome sequences for *T. bauzanensis* DSM22817 and *T. mobilis* KA081020–065 are available at NCBI with respective accession numbers NZ BMDZ01000001.1 and CP003236.1.

## 5. Conclusions

We have developed methods to culture didemnin B (**1**) at >100 mg/L NMR estimated (16 mg/L isolated) levels through a combination of media and growth optimization efforts. These studies indicate that one can direct didemnin production to only afford compound **1,** and to do so in a manner that provides effective chromatographic purification (see NMR data on samples of **1** in CD_3_OD in the Supplementary Information and as tabulated in Table 6). Using 1.7 mm microscaled Atomic Sort [21] analyses, we were able to identify and validate compound purification of small samples (50 µL of extract derived from ~10 mL of culture required) and then translate this into L-scale production. As summarized in Table 5, we observed the production of **1** in cultures of *T. mobilis* JCM21370 and *T. mobilis* YIT12409. While not yet fully optimized, the fact that reproducible conditions were identified with significant titer and isolated yields provides a critical next step to meet the supply demands for **1** and associated one-step oxidation to Aplidine (Plitidepsin).

As part of an ongoing study, we identified biosynthetic gene clusters (BGCs) from the three strains evaluated in this program (Figure 4). In spite of the fact that *T. bauzanensis* TIO7329 was described as producing didemnin [17], analysis of *T. bauzanensis* DSM22817 showed that this was not the case for this strain. Genomic analysis of *T. bauzanensis* DSM22817 revealed the putative didemnin BGC was precisely missing genes for didC–didH and therefore lacks the enzymes to install all but the Gln–Gln–Pyr (efforts were not made to isolate or identify this shunt fragment in extracts of *T. bauzanensis* DSM22817) and Pro–Tyr motifs, which could explain the shunted production of cyclo(Pro–Tyr) (**3**). This suggests that didABIJ or didAB could be used to insert alternative but compatible modules that would generate new chemistry possibilities. The pathways from *T. mobilis* JCM21370 contained didA–didJ, as elucidated in *T. mobilis* KA081020–065 [17]. Efforts are now underway to further explore the genomes of *Tistrella* including *T. mobilis* YIT12409 to guide culture optimization and synthetic biological production, with whole-genome sequence and annotation data to be provided subsequently.

## Figures and Tables

**Table 1:** Antibiotic resistance screen ^a^.

Antibiotic (Concentration)	*T. mobilis* JCM21370	*T. mobilis* YIT12409
apramycin (50 µg/mL)	−	−
carbenicillin (50 µg/mL)	+++	+++
chloramphenicol (25 µg/mL)	−	−
colistin (5 µg/mL)	+++	−
kanamycin (50 µg/mL)	−	−
hygromycin (100 µg/mL)	−	−
nalidixic acid (30 µg/mL)	+	−
none	+++	+++

^a^ Growth given by − (none), + (slight), ++ (reduced), +++ (normal).

**Table 2:** Cultures evaluated in this study.

Culture	Strain ^a^	Media ^b^	V (mL)	T (°C)	Days
A	JCM21370	74NB	200	30	2
B	JCM21370	74NB	5000	30	3
C	YIT12409	74NB	1000	30	3
D	YIT12409	TSB	1000	30	3
E	JCM21370	74NB	100	30	3
F	YIT12409	74NB	100	30	3
G	DSM22817	74NB	100	30	10
H	JCM21370	74NB	2000	30	3
I	YIT12409	74NB	2000	30	3
J	DSM22817	74NB	2000	20	6
K	JCM21370	74NB	1000	30	3
L	YIT12409	74NB	1000	30	3
M	JCM21370	74NB	1000	30	3
N	JCM21370	GYP	1000	30	3
O	JCM21370	SBM	1000	30	3
P	YIT12409	74NB	1000	30	3
Q	YIT12409	GYP	1000	30	3
R	YIT12409	SBM	1000	30	3
S	JCM21370	GYP	2000	30	3

^a^ Strains are listed by code number as given by JCM21370 for *T. mobilis* JCM21370, YIT12409 for *T. mobilis* YIT12409 and DSM22817 for *T. bauzanensis* DSM22817. ^b^ Media is given by 74NB for 74 Nutrient, TSB for Tryptic Soy Broth and GYP for glucose, yeast extract and peptone.

**Table 3:** Atomic Search Analyses of ^1^H–^13^C HSQC spectra from crude *Tistrella* extracts.

Compound	*T. mobilis* JCM21370 (H ^a^)	*T. bauzanensis* DSM22817 (I ^a^)	*T. mobilis* YIT12409 (J ^a^)
methanol	0.009 ^b^, 1/1 ^c^	0.001, 1/1	0.002, 1/1
indole	0.032, 6/6	0.095, 6/6	0.058, 6/6
didemnin B (**1**)	0.056, 51/51	0.587, 5/51 ^d^	0.389, 23/51
cyclo(Pro–Tyr) (**3**)	0.063, 11/11	0.044, 11/11	0.142, 10/11
Leu–Pro–Ile–Pro–Ile (**2**)	0.074, 27/27	0.054, 26/27	0.101, 23/27
cyclo(Ile–Pro–Leu–Pro)	0.183, 19/26	0.175, 21/26	0.154, 17/26
n–hexane	0.136, 2/3	0.123, 3/3	0.121, 2/3

^a^ Letters denote the cultures described in Table 2. ^b^ The first number in each cell is the median Atomic Sort distance ($\tilde{d_{AS}}$). ^c^ The numerator of the fraction is the number of peaks that matched with *d_AS_* ≤ 0.25. The denominator is the total number of peaks for that compound. ^d^ The didemnin B $\tilde{d_{AS}}$ and match fraction for the *T. bauzanensis* DSM22817 strain is included for comparison. The values indicate the match was poor and that **1** was not present.

**Table 4:** Yields of the identified compounds in extract of *Tistrella* cultures.

Database Hit Compound	*T. mobilis* JCM21370 (H ^a^)		*T. bauzanensis* DSM22817 (I ^a^)		*T. mobilis* YIT12409 (J ^a^)	
	[] ^b^, mM	Yield ^c^, µg	[] ^b^, mM	Yield ^c^, µg	[] ^b^, mM	Yield ^c^, µg
indole	51.2	300.7	9.0	53.1	135.4	795.5
didemnin B (**1**)	7.7	427.5	–	–	1.8	102.7
cyclo(Pro–Tyr) (**3**)	22.6	294.2	25.6	333.2	22.7	295.9
Leu–Pro–Ile–Pro–Ile (**2**)	17.5	483.5	18.4	507.5	16.3	449.9
cyclo(Ile–Pro–Leu–Pro)	30.6	643.9	25.7	540.2	28.0	588.9

^a^ Letters denote the cultures described in Table 2. ^b^ Concentrations of each compound in the NMR sample. These values represent an estimated concentration based on NMR integration within the ^1^H NMR spectrum of each sample and do not reflect isolated yields. ^c^ The mass calculations assumed an NMR volume of 50 µL, Leu-Pro-Ile-Pro-Ile (**2**) was the linear pentapeptide with MW = 551.72, and cyclo(Ile–Pro–Leu–Pro) had MW = 420.6. Care was taken to load 50 ± 2 µL into each NMR tube.

**Table 5:** Yields of didemnin B (**1**) in extracts of *T. mobilis* strains JCM21370 and YIT12409 cultures.

Media	*T. mobilis* JCM21370				*T. mobilis* YIT12409			
	Culture ^a^	[] ^b^, mM	Yield ^c^, µg	Titer ^d^, mg/L	Culture ^a^	[] ^b^, mM	Yield ^c^, µg	Titer ^d^, mg/L
74NB	K	7.1	397.3	54.8	L	4.4	247.4	34.1
74NB	M	14.6	809.9	111.7	P	4.7	258.8	35.7
GYP	N	3.7	203.5	28.2	Q	6.8	375.8	51.8
SBM	O	7.1	393.0	54.2	R	5.7	319.0	44.0

^a^ Letters denote the cultures described in Table 2. ^b^ Concentrations of each compound in the NMR sample. These values represent an estimated concentration based on NMR integration within the ^1^H NMR spectrum of each sample and do not reflect isolated yields. ^c^ The mass calculations assumed a volume of 50 µL within the NMR sample. Care was taken to load 50 ± 2 µL into each NMR tube. ^d^ All cultures were conducted at 1 L scale with 2% m/m of the crude extract used for NMR analyses.

**Table 6:** Tabulated NMR data from didemnin B (**1**) in CD_3_OD ^a,b^.

Position	δ ^1^H, Mult (*J* in Hz)	δ^13^C	^1^H, ^1^H–COSY	^1^H, ^13^C–HMBC
Ist1	–	172.9	–	–
Ist2a	3.61, m	40.3	Ist2b	Ist1
Ist2b	2.43, dd (10.4, 17.5)		Ist2a, Ist3	Ist1, Ist3
Ist3	4.05, t (10.5)	67.6	Ist2b	Ist1, Ist4
Ist4	3.97, td (10.3, 2.5)	56.6	IstNH, Ist5	Ist3, Ist6
Ist5	2.00, m	34.9	Ist4, Ist6	Ist7w
Ist6	0.98, d (7.2)	14.5	Ist5, Ist7a, Ist7b	Ist4, Ist7
Ist7a	1.40, m		Ist5, Ist6, Ist7b, Ist8	Ist4w, Ist5w Ist6w, Ist8w
Ist7b	1.26, m		Ist5, Ist6, Ist7a, Ist8	Ist4w, Ist5w, Ist6w, Ist8w
Ist8	0.97, d (6.6)	12.1	Ist7	–
IstNH	7.35, d (10.0)	–	Ist4	Thr1w
Hip1	–	171.3	–	–
Hip2	4.12, q (6.7)	49.2	Hip3	Hip1, Hip3, Hip4
Hip3	1.30, d (6.9)	15.9	Hip2	Hip1, Hip2, Hip4
Hip4	–	205.7	–	–
Hip5	5.05, d (4.0)	81.9	Hip6	Hip4, Hip6, Hip7, Hip8w, Ist1
Hip6	2.32, m	31.0	Hip5, Hip7, Hip8	Hip8w
Hip7	0.87, d (6.9)	17.0	Hip6	Hip5, Hip6, Hip8
Hip8	0.93, d (6.8)	19.2	Hip6	Hip5, Hip6, Hip7
Leu1	–	171.5	–	–
Leu2	4.78, m	50.6	LeuNH, Leu3a, Leu3b	–
Leu3a	1.64, m	42.2	Leu2, Leu3b	–
Leu3b	1.23, m		Leu2, Leu3a	–
Leu4	1.52, m	25.7	Leu3a, Leu3b, Leu5, Leu6	–
Leu5	0.92, d (6.7)	21.2	Leu4	Leu3, Leu4, Leu6
Leu6	0.96, d (6.9)	23.8	Leu4	Leu3, Leu4, Leu5
LeuNH	8.1, d (9.2)	–	Leu2	Hip1
Pro1	–	172.5	–	–
Pro2	4.80, m	58.4	Pro3a, Pro3b	–
Pro3a	2.19, m	28.7	Pro2, Pro3b	Pro1, Pro2w
Pro3b	1.70, dt (12.3, 6.1)		Pro2, Pro3a	Pro1, Pro5
Pro4	2.06, m	25.5	Pro5a, Pro5b	Pro2, Pro5
Pro5a	3.74, dt (9.7, 6.8)	47.9	Pro4	–
Pro5b	3.58, dt (5.3, 3.1)		Pro4	Pro1
MTyr1	–	170.0	–	–
MTyr2	4.01. dd (10.6, 4.7)	66.3	MTyr3a, MTyr3b	MTyr1, MTyr3, MTyrNM, Pro1
MTyr3a	3.15, dd (14.1, 10.7)	34.6	MTyr2, MTyr3b	MTyr1w, MTyr2, MTyr4, MTyr5
MTyr3b	3.29, m		MTyr2, MTyr3a	MTyr2, MTyr4, MTyr5
MTyr4	–	130.6	–	–
MTyr5	7.14, d (8.6)	131.8	MTyr6	MTyr3, MTyr5, MTyr6, MTyr7
MTyr6	6.88, d (8.5)	114.7	MTyr5	MTry4, MTyr6, MTyr7
MTyr7	–	159.8	–	–
MTyr8	3.78, s	55.3		MTyr7
MTyrNMe	2.62, s	39.1	–	–
Thr1	–	171.1	–	–
Thr2	4.54, dd (5.1, 2.4)	59.1	ThrNH	Thr3, Thr4w
Thr3	5.37, d (5.9)	71.1	Thr4	Thr2w, MTyr1
Thr4	1.35, d (6.3)	16.6	Thr3	Thr2, Thr3
ThrNH	7.94, d (4.9)	–	Thr2	MLeu1
MLeu1	–	173.0	–	–
MLeu2	5.39, d (8.2)	55.9	MLeu3	HPro1, MLeu1, MLeu3, MeLeu4, MLeuNMe
MLeu3	1.80, dd (8.5, 6.9)	37.0	MLeu2, MLeu4	HPro1, MLeu1, MLeu2, MLeu4, MLeu5, MLeu6
MLeu4	1.47, m	25.5	MLeu3, MLeu5, MLeu6	MLeu3, MLeu4, MLeu5
MLeu5	0.89, d (6.5)	21.3	MLeu4	MLeu3, MLeu4, MLeu6
MLeu6	0.95, d (6.7)	23.8	MLeu4	MLeu3, MLeu4, MLeu5
MLeuNMe	3.19, s	31.5	–	MLeu2, HPro1
HPro1	–	175.4	–	–
HPro2	4.84, m	58.0	HPro3a, HPro3b	HPro3
HPro3a	2.28, m	29.2	HPro2, HPro3b	HPro5w
HPro3b	1.85, m		HPro2, HPro3a	HPro1w
HPro4a	2.16, m	26.6	HPro4b, HPro5a, HPro5b	–
HPro4b	2.03, m		HPro4a, HPro5a, HPro5b	HPro2, HPro3, HPro5
HPro5a	3.88, ddd (10.8, 7.3, 3.6)	48.1	HPro4a, HPro4b, HPro5b	HPro4w
HPro5b	3.64, dd (10.0, 6.6)		HPro4a, HPro4b, HPro5a	–
HP1	–	174.1	–	–
HP2	4.47, q (6.6)	67.3	HP3	HP1, HP3
HP3	1.38, d (6.6)	19.9	HP2	HP1, HP2

^a^ Abbreviations: MTyr = *N*–methylTyr, MLeu = *N*–methylLeu, SHP = *N–(S*)–2–hydroxypropanoyl. ^b^ Coupling abbreviations are given by s = singlet, d = doublet, t = triplet, q = quartet and m = multiplet.

## Data Availability

Copies of raw NMR spectra can be provided by email request to J.J.L.C. at i@xenobe.org or jlaclair@ucsd.edu.

## Supplementary Materials

The following information can be downloaded at: https://www.mdpi.com/article/10.3390/md21020056/s1. Copies of NMR data and Atomic Sort results.
