# Supplementary material for: A Survey of Didemnin Depsipeptide Production in Tistrella
Source: Mar Drugs. 2023 Jan 17;21(2):56. doi: 10.3390/md21020056 (PMC9964501; doi:10.3390/md21020056)

SUPPORTING INFORMATION

A Survey of Didemnin Depsipeptide Production in *Tistrella*

Robert J. Stankey,[1] Don Johnson, [1] Brendan M. Duggan,[2]David A. Mead[1,*] and James J. La Clair, [3,4,*]

[1] Terra Bioworks Inc., Middleton, Wisconsin, 53562, United States

[2] Skaggs School of Pharmacy and Pharmaceutical Sciences, University of California, San Diego, 9500 Gilman Drive, La Jolla, California, 92093, United States

[3] Department of Chemistry and Biochemistry, University of California at San Diego, La Jolla, California, 92093-0358, United States

[4] Xenobe Research Institute, P. O. Box 3052, San Diego, CA 92163-1052

***** Correspondence: dmead@teraabioworks.com (D.A.M.) or i@xenobe.org (J.J.L.)

^{1}H-NMR (600 MHz) spectrum of didemnin B in CD_3OD

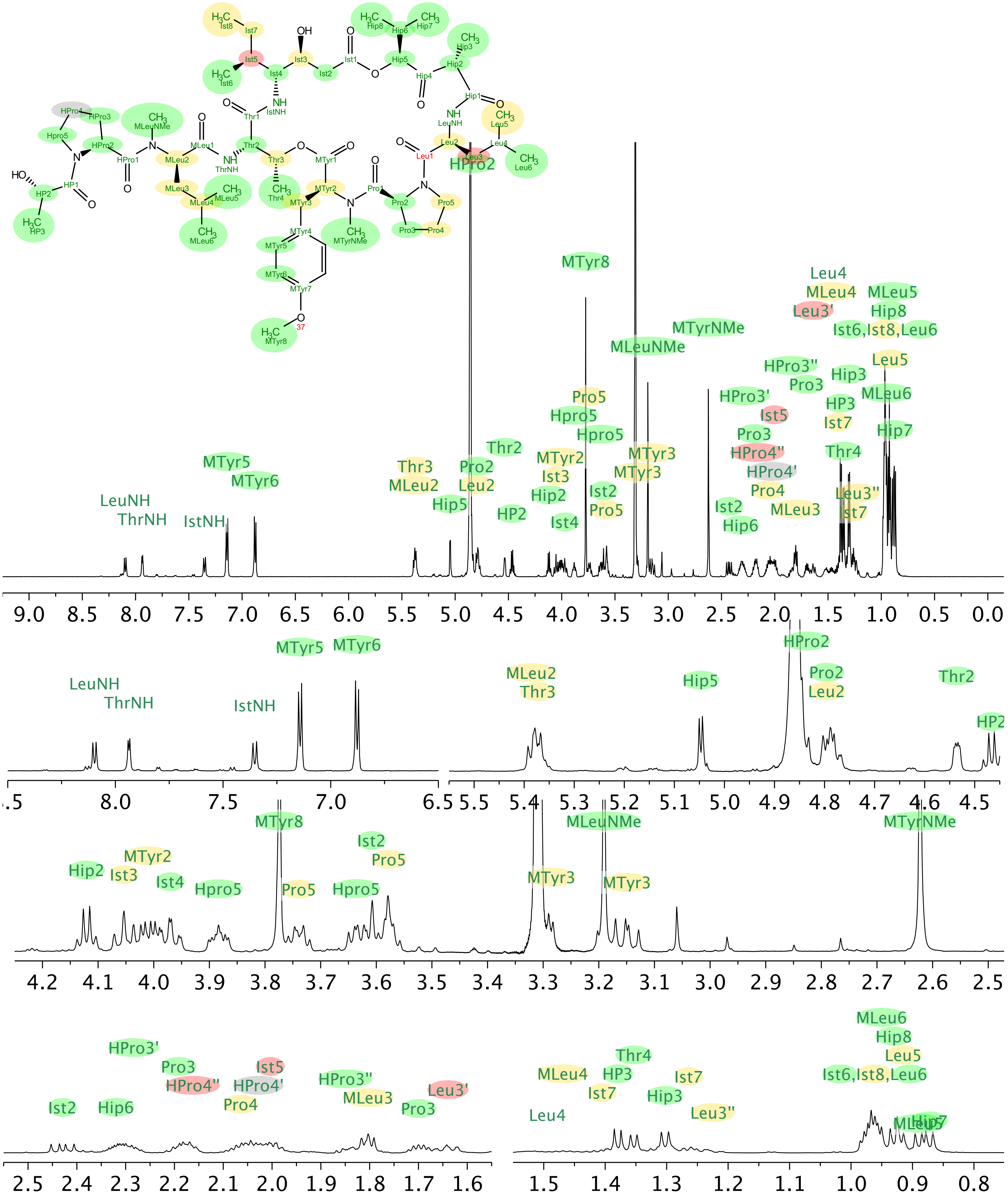

$^{1}H,^{1}H$-COSY (600 MHz) spectrum of didemnin B in CD_3OD

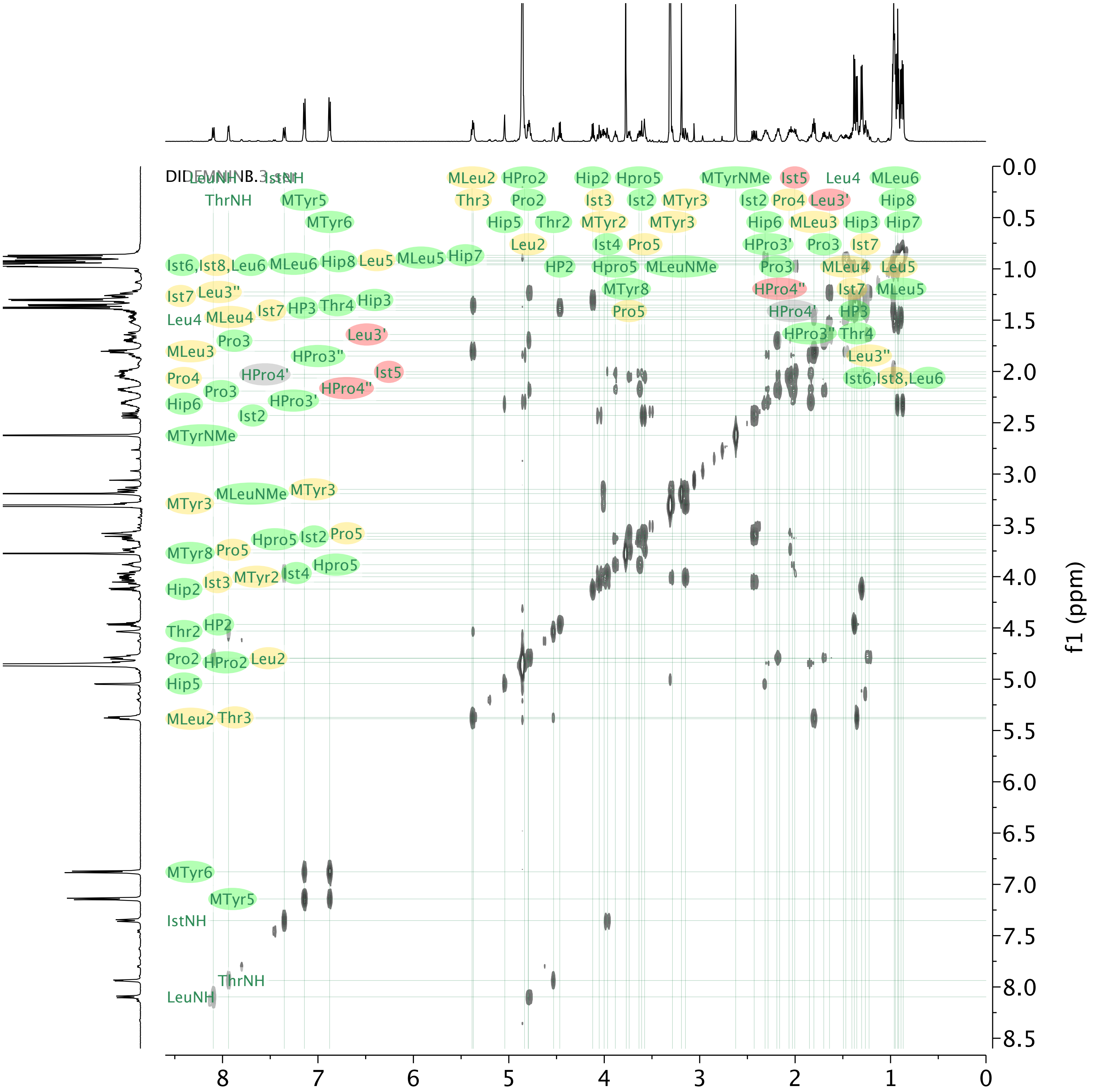

$^{1}H,^{13}C$-HSQC (600 MHz) spectrum of didemnin B in CD_3OD

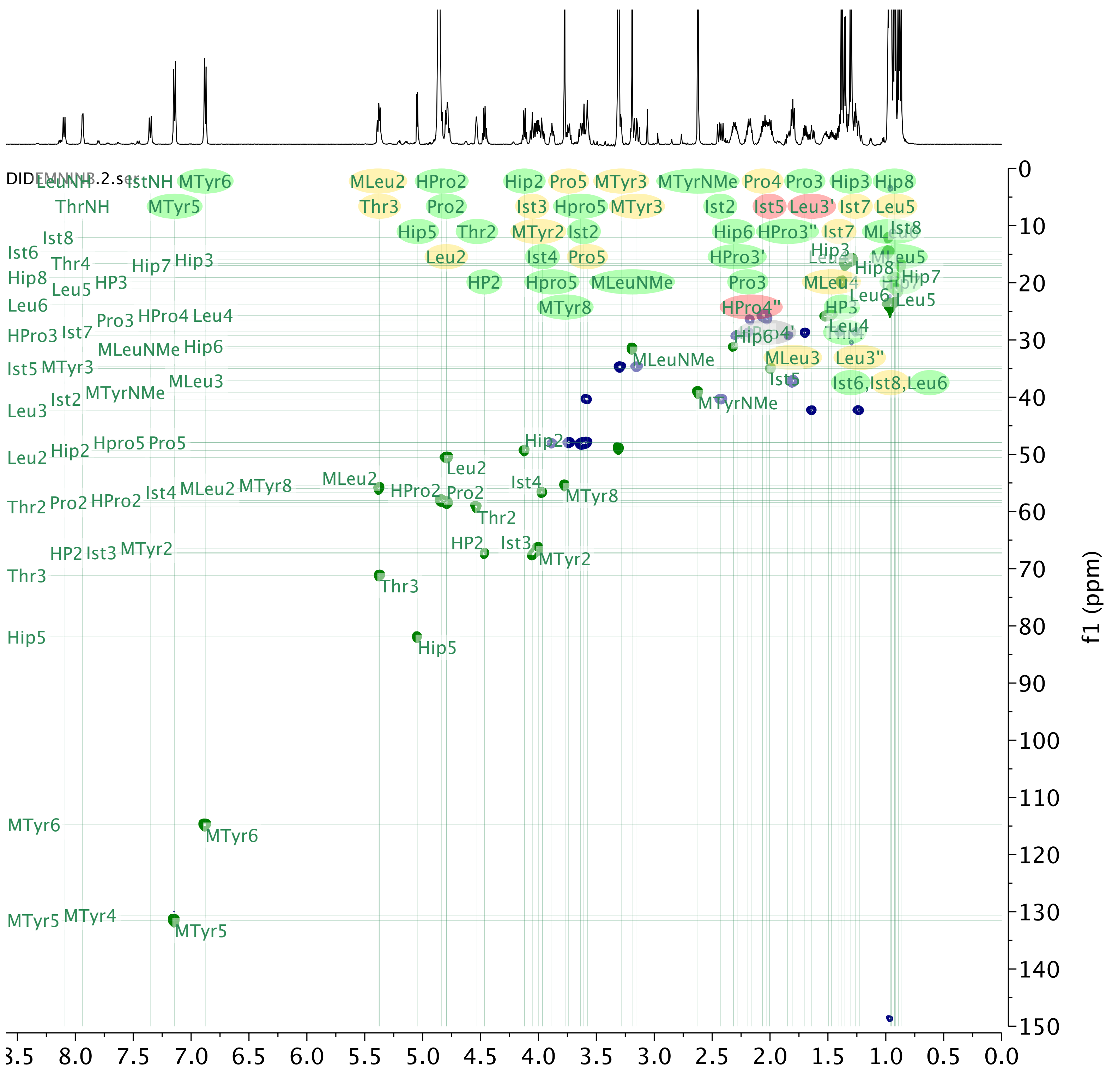

$^{1}H,^{13}C$-HMBC (600 MHz) spectrum of didemnin B in CD_3OD

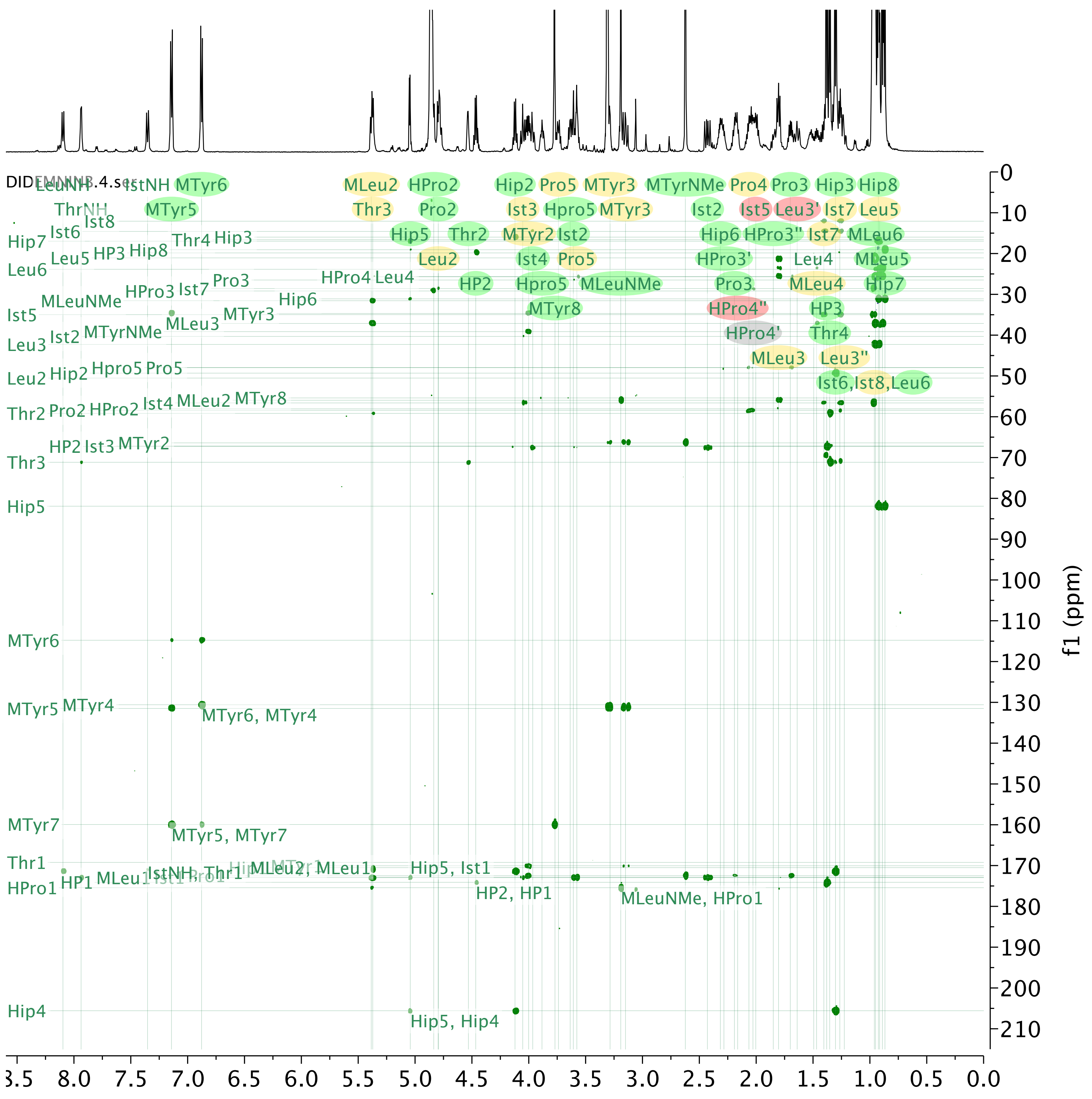

^{1}H-NMR (600 MHz) spectra of extracts from cultures **K - R** as compared to didemnin B in CD_3OD

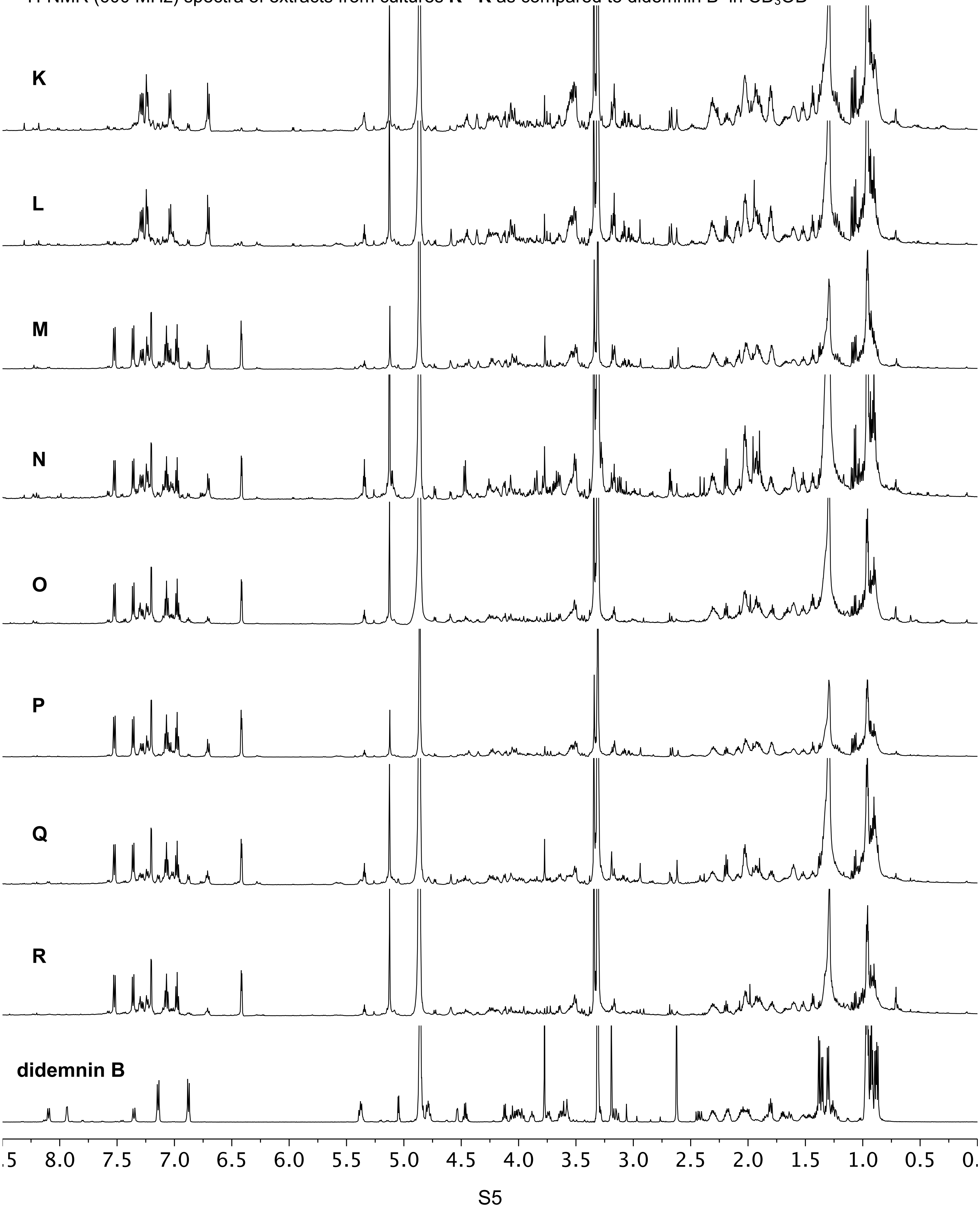

^{1}H-NMR (600 MHz) spectra of extracts from cultures **K - R** as compared to didemnin B in CD_3OD

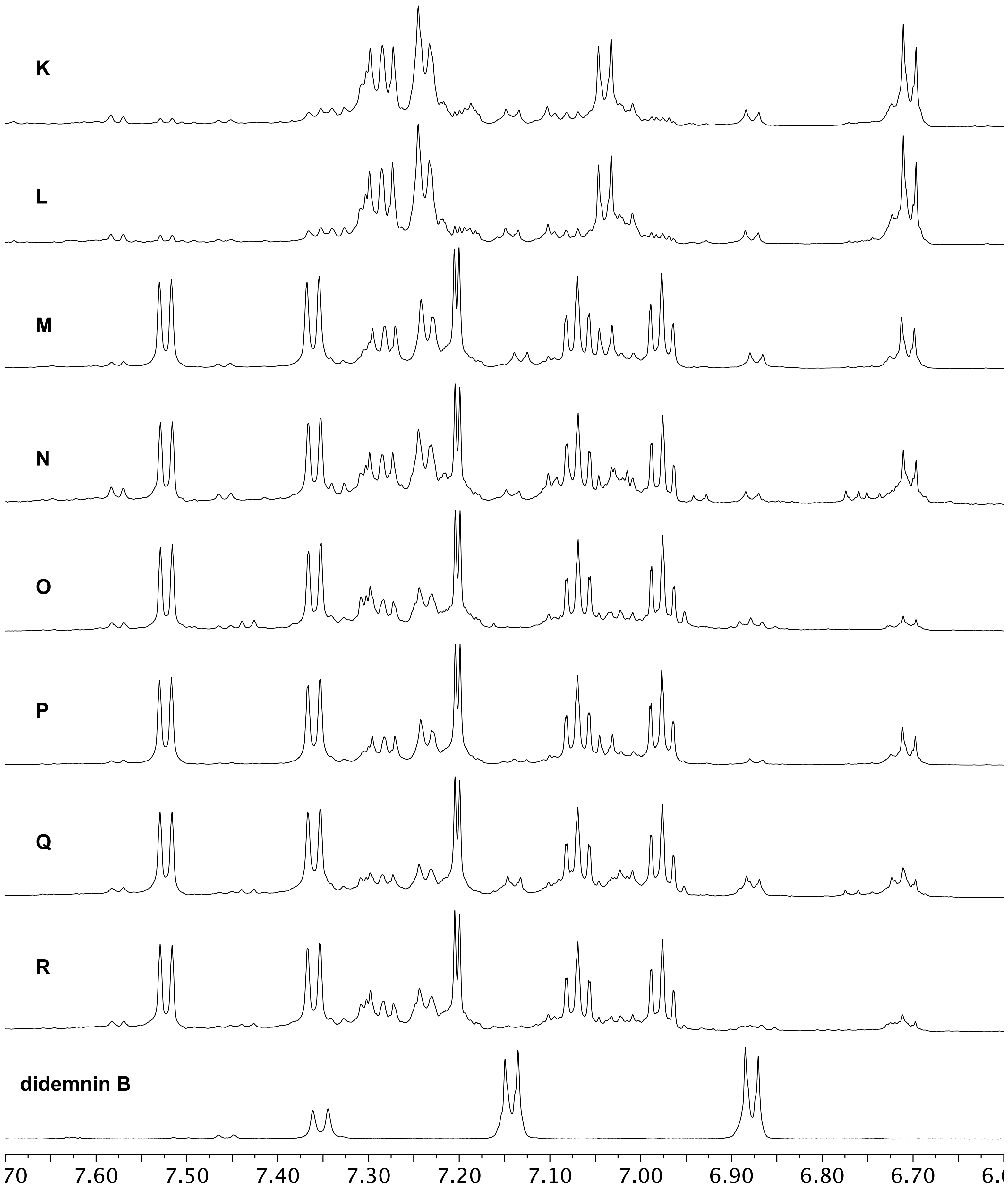

^{1}H-NMR (600 MHz) spectra of extracts from cultures **K - R** as compared to didemnin B in CD_3OD

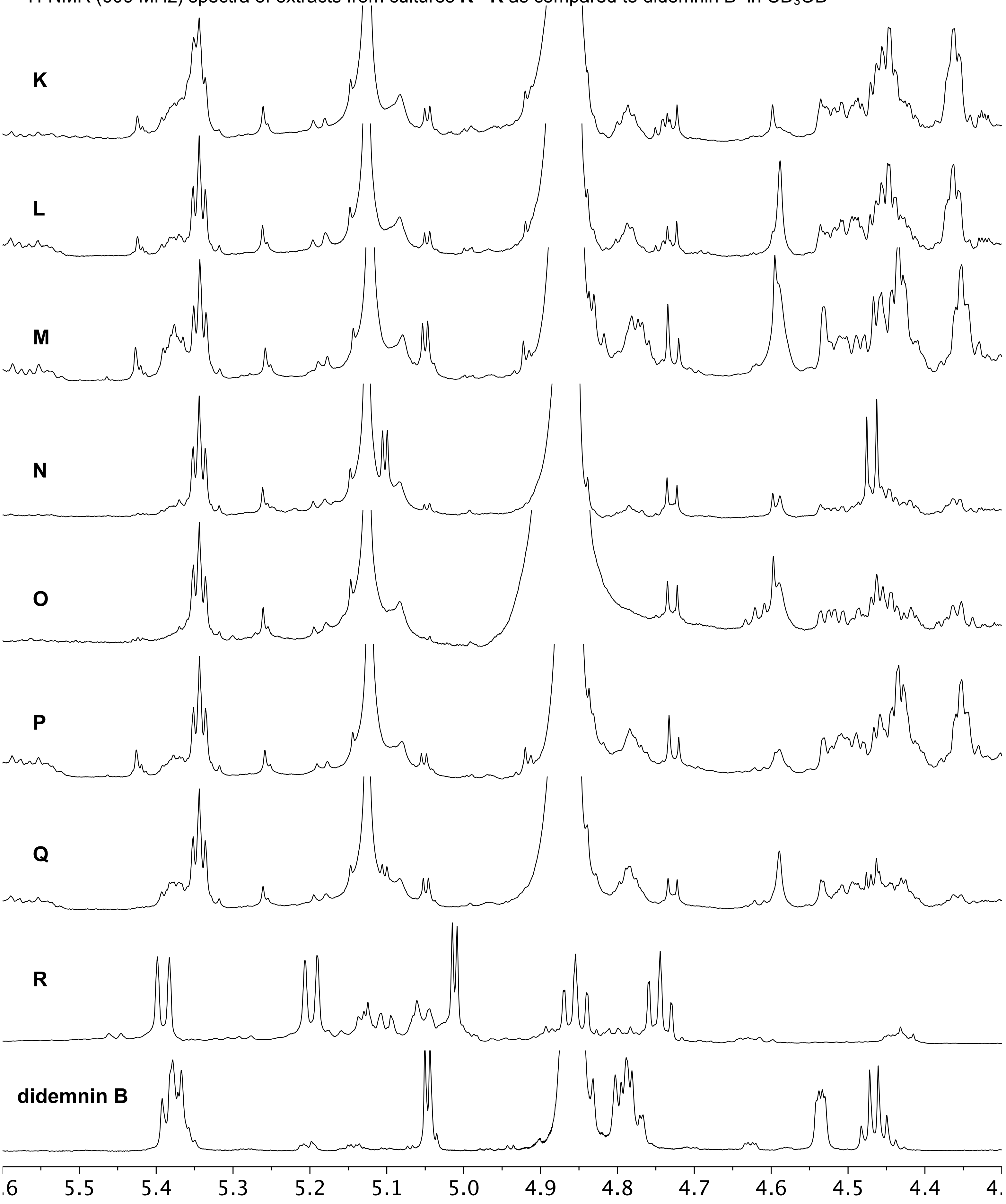

^{1}H-NMR (600 MHz) spectra of extracts from cultures **K - R** as compared to didemnin B in CD_3OD

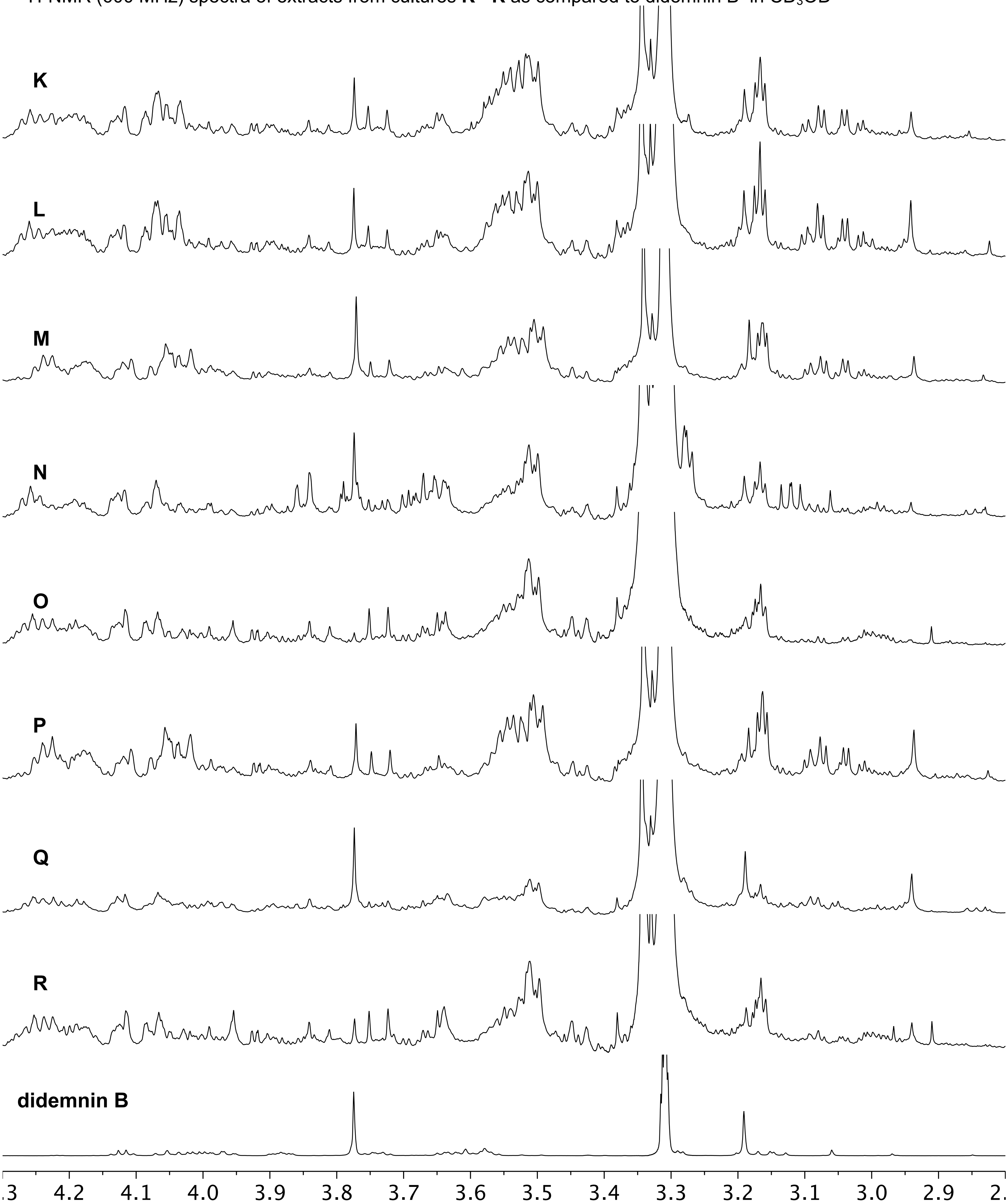

^{1}H-NMR (600 MHz) spectra of extracts from cultures **K - R** as compared to didemnin B in CD_3OD

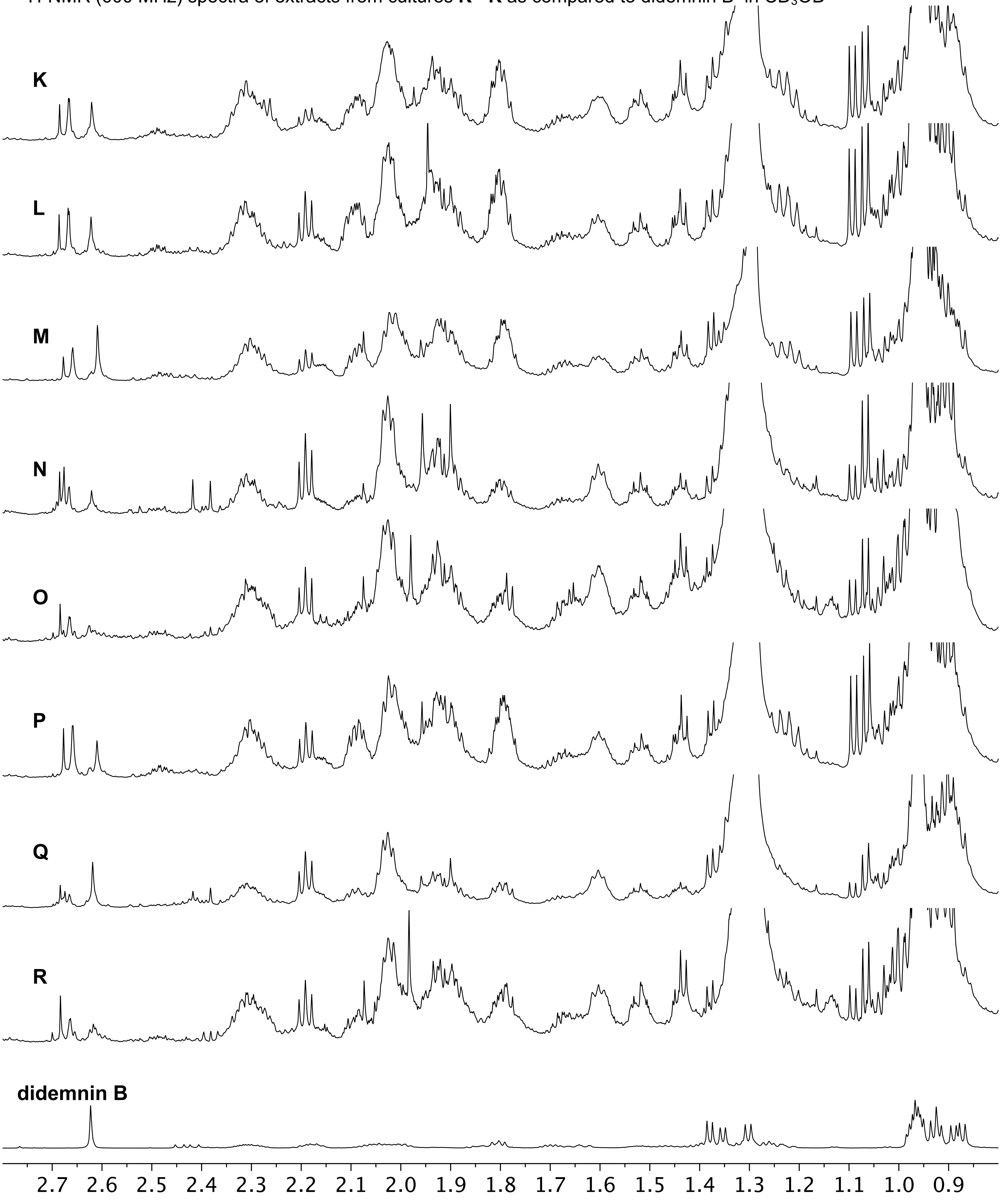

Supplement: Supplementary file 1 [file marinedrugs-21-00056-s001.zip › MARINEDRUGS_DIDEMNIN_NMR_57.pdf]
